# *In vitro* Activity of Apramycin Against Carbapenem-Resistant and Hypervirulent *Klebsiella pneumoniae* Isolates

**DOI:** 10.3389/fmicb.2020.00425

**Published:** 2020-03-13

**Authors:** Mingju Hao, Xiaohong Shi, Jingnan Lv, Siqiang Niu, Shiqing Cheng, Hong Du, Fangyou Yu, Yi-Wei Tang, Barry N. Kreiswirth, Haifang Zhang, Liang Chen

**Affiliations:** ^1^Department of Laboratory Medicine, Shandong Provincial Qianfoshan Hospital, The First Hospital Affiliated With Shandong First Medical University, Jinan, China; ^2^Department of Clinical Laboratory, The Second Affiliated Hospital of Soochow University, Suzhou, China; ^3^Department of Laboratory Medicine, The First Affiliated Hospital of Chongqing Medical University, Chongqing, China; ^4^Department of Laboratory Medicine, Shandong Provincial Hospital Affiliated to Shandong University, Jinan, China; ^5^Department of Clinical Laboratory, Shanghai Pulmonary Hospital, Tongji University School of Medicine, Shanghai, China; ^6^Department of Laboratory Medicine, Memorial Sloan Kettering Cancer Center; Department of Pathology and Laboratory Medicine, Weill Medical College of Cornell University, New York, NY, United States; ^7^Hackensack-Meridian Health Center for Discovery and Innovation, Nutley, NJ, United States

**Keywords:** apramycin, susceptibility, carbapenem resistance, hypervirulent *Klebsiella pneumoniae*, aminoglycoside

## Abstract

**Objective:**

The emergence of carbapenem-resistant and hypervirulent *Klebsiella pneumoniae* (CR-hvKp) strains poses a significant public threat, and effective antimicrobial therapy is urgently needed. Recent studies indicated that apramycin is a potent antibiotic with good activity against a range of multi-drug resistant pathogens. In this study, we evaluated the *in vitro* activity of apramycin against clinical CR-hvKp along with carbapenem-resistant non-hvKp (CR-non-hvKp) isolates.

**Methods:**

Broth microdilution method was used to evaluate the *in vitro* activities of apramycin, gentamicin, amikacin, imipenem, meropenem, doripenem, ertapenem and other comparator “last-resort” antimicrobial agents, including ceftazidime-avibactam, colistin and tigecycline, against eighty-four CR-hvKp and forty CR-non-hvKp isolates collected from three Chinese hospitals. Multilocus Sequence typing (MLST), molecular capsule typing (*wzi* sequencing) and antimicrobial resistance genes were examined by PCR and Sanger sequencing. Pulsed-field gel electrophoresis and next generation sequencing were conducted on selected isolates.

**Results:**

Among the 84 CR-hvKp isolates, 97.6, 100, 97.6, and 100% were resistant to imipenem, meropenem, doripenem and ertapenem, respectively. Apramycin demonstrated an MIC_50_/MIC_90_ of 4/8 μg/mL against the CR-hvKp isolates. In contrast, the MIC_50_/MIC_90_ for amikacin and gentamicin were >64/>64 μg/mL. All CR-hvKp isolates were susceptible to ceftazidime-avibactam, colistin and tigecycline with the MIC_50_/MIC_90_ values of 0.5/1, 0.25/0.5, 1/1, respectively. For CR-non-hvKp, The MIC_50/90_ values for apramycin, gentamicin and amikacin were 2/8, >64/>64, and >64/>64 μg/mL, respectively. There were no statistical significance in the resistance rates of antimicrobial agents between CR-hvKp and CR-non-hvKp groups (*p* > 0.05). Genetic analysis revealed that all CR-hvKp isolates harbored *bla*_KPC–2_, and 94% (*n* = 79) belong to the ST11 high-risk clone. 93.6% (44/47) of amikacin or gentamicin resistant strains carried 16S rRNA methyltransferases gene *rmtB*.

**Conclusion:**

Apramycin demonstrated potent *in vitro* activity against CR-hvKp isolates, including those were resistant to amikacin or gentamicin. Further studies are needed to evaluate the applicability of apramycin to be used as a therapeutic antibiotic against CR-hvKp infections.

## Introduction

*Klebsiella pneumoniae* is a clinically important pathogen, causing a wide range of diseases including pneumonia, urinary tract infections (UTIs), bloodstream infections, in hospital settings among neonates, elderly and immunocompromised individuals (Paczosa and Mecsas, 2016; Bengoechea and Sa Pessoa, 2019). Hypervirulent K. pneumoniae (hvKp) is an increasing recognized pathotype that is more virulent than classical K. pneumoniae (cKp), which showed the propensity to cause serious, life-threatening infections in otherwise healthy individuals in the community (Shon et al., 2013; Gu et al., 2018).

Historically, hvKp isolates are susceptible to most antibiotics, however, multidrug-resistant hvKp, especially carbapenem-resistant strains, are increasingly reported in the past few years (Cejas et al., 2014; Li et al., 2014; Siu et al., 2014). The emergence of carbapenem-resistant and hypervirulent *K. pneumoniae* (CR-hvKp) was either due to the acquisition of a pLVPK-like virulence plasmid by classic carbapenem-resistant *K. pneumoniae* strains (Gu et al., 2018) or through acquisition of a carbapenemase-encoding plasmid by hvKp isolates (Shon et al., 2013). The rmpA, rmpA2, iutA, iucABCD and iroBCDN genes harbored by pLVPK-like virulence plasmid is responsible for the virulence phenotype (Yang et al., 2019). A recent study from China reported a fatal outbreak of ventilator associated pneumonia caused by ST11 CR-hvKp strains (Gu et al., 2018). More recently, one hypermucoviscos isolate with KPC-3 carbapenemase-encoding plasmid was firstly described in the United States, which also exhibited colistin heteroresistance (Wozniak et al., 2019). The rapid dissemination of CR-hvKp strains in different global regions underscores the urgent need of appropriate antibiotic therapy against these life-threating pathogens.

Aminoglycosides including amikacin, gentamicin and tobramycin, which belong to the 4,6-disubstituted deoxystreptamine (DOS) subclass, are among the few drugs that retain certain *in vitro* activity against CRE (Livermore et al., 2011). However, the acquisition of 16S rRNA methylases confer high level resistance to the 4,6-disubstituted DOS aminoglycosides, which imposes serious challenge for clinical management (Hu et al., 2017). Apramycin is of the 4-monosubstituted DOS subclass, markedly different in its chemical structure from other clinically used aminoglycoside antibiotics. Its unique structure makes apramycin molecules inherently resilient to almost all resistance determinants commonly found in multi-drug resistant strains (Juhas et al., 2019). Indeed, recent studies indicated that apramycin is a potent antibiotic with good activity against a range of clinical pathogens, including multidrug-resistant *Mycobacterium tuberculosis*, carbapenem-resistant *Enterobacteriaceae*, spectinomycin-resistant *Neisseria gonorrhoeae* and *Staphylococcus aureus* (Smith and Kirby, 2016; Hu et al., 2017; Truelson et al., 2018; Galani et al., 2019; Riedel et al., 2019). In order to evaluate whether apramycin can be served as an alternative antibiotic to treat the CR-hvKp infections, we examined the *in vitro* activity of apramycin against a collection of CR-hvKp isolates collected from three different hospitals in China. Its *in vitro* activity was compared with the results from other aminoglycosides (amikacin and gentamicin) and some “last resort” antibiotics, including colistin, tigecycline and ceftazidime-avibactam.

## Materials and Methods

### Bacterial Isolates

Eighty-four unique (one isolate per patient) CR-hvKp isolates, including 65 isolates from our previous study (Yu et al., 2018a), were included. They were collected from three hospitals across different regions in China (Shandong, Jiangsu and Shanghai) between 2014 and 2019. These isolates were obtained from sputum (53.6%, 45/84), urine (14.3%, 12/84), blood (14.3%, 12/84), pus (4.8%, 4/84), ascites (3.6%, 3/84) and other sources (9.5%, 8/84). In this study, we defined an hvKp strain based upon demonstration of a positive string test (hypermucoviscosity) and co-harboring the genes *rmpA* (*rmpA* or *rmpA2*) and *iutA* as previously (Yu et al., 2018a). All 84 isolates were hypermucoviscous (string test >5 mm) and were positive for the *rmpA/rmpA2* and *iutA* by PCR analysis (Yu et al., 2018b). In addition, 40 unique strains of carbapenem resistant non-hypervirulent *K. pneumoniae* (CR-non-hvKp) isolates, with negative hypermucoviscous phenotype and negative *rmpA/rmpA2* and *iutA* PCR results, were collected from the same three hospitals, and were included to compare with the susceptibility results of CR-hvKp.

### Antibiotic Susceptibility Assay

The antimicrobial susceptibility was assessed *via* a minimum inhibitory concentration (MIC) broth microdilution method. Results were interpreted using Clinical Laboratory Standards Institute (CLSI) breakpoints (Clinical and Laboratory Standards Institute, 2019) with the exception of tigecycline and polymyxin B, which were interpreted using EUCAST breakpoints (The European Committee on Antimicrobial Susceptibility Testing, 2019). National Antimicrobial Resistance Monitoring System (NARMS) breakpoints for enteric bacteria were used for the interpretation of apramycin susceptibility, in which strains were classified as susceptible (MIC ≤ 8 μg/mL), intermediate (MIC = 16∼32 μg/mL), or resistant (MIC ≥ 64 μg/mL) (National Antibiotic Resistance Monitoring System [NARMS], 2002). MICs were performed in duplicate on two separate days. *Escherichia coli* ATCC 25922 and *Pseudomonas aeruginosa* ATCC 27853 were used as the quality control strains in each experiment. *K. pneumoniae* ATCC 700603 was used as quality control strain for ceftazidime-avibactam which was tested against ceftazidime-avibactam and ceftazidime alone to confirm the activity of avibactam in the combination.

### Molecular Typing and Screening of Antibiotic Resistance Genes

All CR-Kp isolates including CR-hvKp and CR-non-hvKp were genotyped by multilocus Sequence Typing (MLST) and *wzi* sequencing (Brisse et al., 2013). Pulsed-field gel electrophoresis (PFGE) of Xbal-digested genomic DNA samples of 15 selected CR-hvKp isolates was performed with a CHEF MAPPER XA apparatus (Bio-Rad, United States). PFGE patterns were analyzed using GelJ software 2.0v (Heras et al., 2015).

A multiplex PCR was used to detect KPC, NDM, VIM, IMP, and OXA-48-like carbapenemase genes, followed by Sanger sequencing (Chen et al., 2011; Chavda et al., 2016; Evans et al., 2016). Common aminoglycoside modifying enzyme (AME) and RMT-encoding genes were determined by PCR and Sanger sequencing. AME coding genes, including *aac(3*′*)-IIa, aac(3*′*)-IId, aac(3*′*)-IV, aac(6*′*)-IIa, aph(3*′*)-Ia, aph(3*′*)-Ib, ant(2*′*)-Ia* and *ant(3*′*)-Ia*, and ARM/RMT methyltransferases coding genes, including *armA, rmtA, rmtB, rmtC, rmtD* and *npmA* (Wu et al., 2009), were examined. The primers used in this study were listed in Supplementary Table S1.

The apramycin resistant isolate, KpApr172, was subject to next generation sequencing using the Illumina HiSeq system (Illumina, San Diego, CA, United States). Genomic DNA was isolated using a Wizard^®^ Genomic DNA Purification Kit (Promega, Madison, WI, United States). Sequencing reads were *de novo* assembled using Spades 3.12.0 (Bankevich et al., 2012). *In silico* multi-locus sequence typing was performed using MLST 2.0 (Larsen et al., 2012) while the acquired antimicrobial resistance genes were identified using ResFinder 3.0 (Zankari et al., 2012). The plasmid replicons in the sequenced isolates were identified using PlasmidFinder 2.0 (Carattoli et al., 2014). The whole-genome sequence of KpApr172 was deposited in the GenBank database under accession number WUJI00000000.

### Statistical Analysis

Chi-square and Fisher’s exact analysis were employed to compare resistance rate of antimicrobial agents between CR-hvKp and CR-non-hvKp groups. Statistical analyses were performed using GraphPad Prism 6.0 (GraphPad Software, La Jolla, CA, United States). *P* value of <0.05 was considered to indicate statistically significant differences.

## Results

### Susceptibility Testing

**FIGURE 1:**
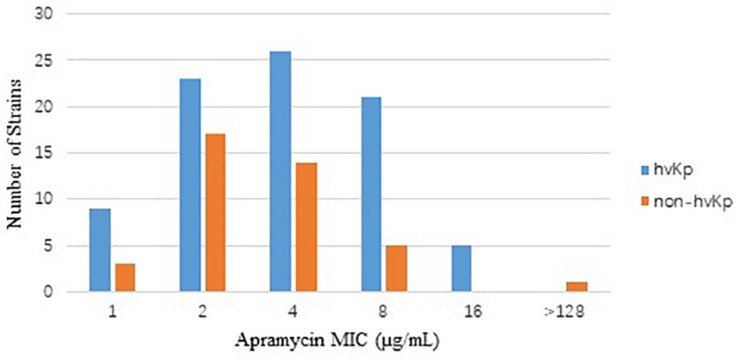
Distribution of apramycin MIC in CR-hvKp (*n* = 84) and CR-non-hvKp (*n* = 40) isolates. hvKp: hypervirulent *Klebsiella pneumonia*, non-hvKp: non-hypervirulent *Klebsiella pneumonia*.

The *in vitro* activities of apramycin and other antimicrobials against 84 clinical isolates CR-hvKp and 40 CR-non-hvKp strains were summarized in Table 1. The CR-hvKp isolates showed high-level resistance to four tested carbapenems, with MIC_50/90_ values 128/>256, 128/> 256, 64/128 and 128/>256 μg/mL for meropenem, imipenem, doripenem, and ertapenem, respectively, 97.6, 100, 97.6, and 100% of the isolates were resistant to imipenem, meropenem, doripenem and ertapenem. All isolates were resistant to ceftazidime and 97.6% (*n* = 82) isolates were resistant to aztreonam. Apramycin exhibited significantly better antimicrobial activity than the clinical standard-of-care aminoglycosides gentamicin and amikacin, with MIC_50/90_ values 4/8 μg/mL). The MIC ranged from 1 to 16 μg/mL (Figure 1). By contrast, the MIC_50__/90_ values for gentamicin and amikacin were both >64/>64 μg/mL.

**TABLE 1:** *In vitro* susceptibility of different antibiotics against 84 CR-hvKp and 40 CR-non-hvKp isolates.

Antimicrobial agents	MIC (range)	Resistance	MIC_50_	MIC_90_
	(μg/mL)	n (%)	(μg/mL)	(μg/mL)
**CR-hvKp isolates (*n* = 84)**				
Meropenem	4 to >256	84 (100)	128	>256
Imipenem	2 to >256	82 (97.6)	128	>256
Doripenem	1 to >256	82 (97.6)	64	128
Ertapenem	2 to >256	84 (100)	128	>256
Aztreonam	8 to >64	82 (97.6)	>64	>64
Ceftazidime	16 to >256	84(100)	>256	>256
Ceftazidime-avibactam	<0.125 to 0.25	0 (0)	0.5	1
Colistin	<0.125 to 1	0 (0)	0.25	0.5
Tigecycline	0.5 to 1	0 (0)	1	1
Gentamicin	<0.5 to >64	47(55.9)	>64	>64
Amikacin	1 to >64	46 (54.8)	>64	>64
Apramycin	1 to 16	0 (0)	4	8
**CR-non-hvKp isolates (*n* = 40)**				
Meropenem	2 to >256	39 (97.5)	128	>256
Imipenem	2 to >256	39 (97.5)	128	>256
Doripenem	1 to >256	39 (97.5)	128	128
Ertapenem	16 to >256	40 (100)	256	>256
Aztreonam	>64	40(100)	>64	>64
Ceftazidime	32 to >256	40(100)	>256	>256
Ceftazidime-avibactam	< 0.125 to >128	5 (12.5)	0.5	>128
Colistin	<0.125 to 1	0 (0)	0.25	0.5
Tigecycline	0.5 to 1	0 (0)	1	1
Gentamicin	<0.5 to >64	22(55)	>64	>64
Amikacin	2 to >64	23 (57.5)	>64	>64
Apramycin	1 to >128	1 (2.5)	2	8

For CR-non-hvKp, 2.5, 55, and 57.5% of the isolates were resistant to apramycin, gentamicin and amikacin, respectively. The MIC_50/90_ values for apramycin, gentamicin and amikacin were 2/8, >64/>64, and >64/> 4 μg/mL, respectively. One isolate was resistant to all three aminoglycosides including apramycin (MIC > 128 μg/mL). Five NDM producing CR-non-hvKp isolates were resistant to ceftazidime-avibactam (MIC > 128 μg/mL), but they remained susceptible to colistin and tigecycline. There were no statistical significance in the resistance rates of antimicrobial agents between CR-hvKp and CR-non-hvKp groups (*p* > 0.05).

### Molecular Typing and *wzi* Genes Sequencing

Multilocus sequence typing showed that most clinical CR-hvKp strains (94%, 79/84) belong to ST11, the most prevalent CR-Kp ST in China (Gu et al., 2018), while the remaining five isolates were from ST268 (*n* = 2), ST65 (*n* = 1), ST412 (*n* = 1), and ST595 (*n* = 1). Among the 79 ST11 isolates, 44 have the capsular polysaccharide *wzi* allele 64 (*wzi64*) (corresponding with capsular type KL64), and the other 35 isolates carry *wzi209* (corresponding with capsular KL47). For CR-non-hvKp strains, 70% (28/40) of the isolates were identified as ST11, carrying *wzi64* (*n* = 18) and *wzi209* (*n* = 10), respectively. The other 12 CR-non-hvKp isolates were from ST2407 (*n* = 3), ST1308 (*n* = 3), ST25 (*n* = 2), ST307 (*n* = 1), ST1308 (*n* = 1), ST412 (*n* = 1), and ST48 (*n* = 1). To further explore the relatedness of individual CR-hvKp strains, we randomly selected 15 ST11 CR-hvKp strains from the three hospitals for PFGE analysis (Supplementary Figure S1). The results showed these isolates generally clustered by hospitals and capsular KL types, but displayed various PFGE profiles. Our results suggested that apramycin is actively against CR-hvKp and CR-non-hvKp isolates most prevalent STs from different geographical regions (Jiangsu, Shanghai, and Shandong) in China.

### Genotyping of Carbapenemase and Aminoglycoside Resistance Genes

The distribution of carbapenemase and aminoglycoside resistance genes were summarized in Table 2. All CR-hvKp isolates and 72.5% (29/40) of CR-non-hvKp isolates harbored *bla*_KPC–2_. Five CR-non-hvKp strains harbored *bla*_NDM–1_ (*n* = 4) and *bla*_NDM–5_ (*n* = 1). 93.6% (44/47) of the CR-hvKp and 95.6% (22/23) of the CR-non-hvKp isolates that were resistant to amikacin or gentamicin carried the RMT gene *rmtB*.

**TABLE 2:** Prevalence of carbapenemase and aminoglycoside resistance genes in CR-hvKp and CR-non-hvKp isolates.

Resistance genes		CR-hvKp	CR-non-hvKp
		(*n* = 84)	(*n* = 40)
Carbapenemase	KPC-2	84(100%)	29(72.5%)
	NDM	0(0%)	5(12.5%)
	IMP	0(0%)	0(0%)
	VIM	0(0%)	0(0%)
	OXA-48	0(0%)	0(0%)
Aminoglycoside modifying enzyme (AME)	*Aac(3*′*)-IIa*	1(1.2%)	2(5%)
	*Aac(3*′*)-IId*	1(1.2%)	0(0%)
	*Aac(3*′*)-IV*	0(0%)	1(2.5%)
	*Aac(6*′*)-Ib*	6(7.14%)	3(7.5%)
	*Aac(6*′*)-IIa*	0(0%)	0(0%)
	*Aph(3*′*)-Ia*	4(4.76%)	9(22.5%)
	*Aph(3*′*)-Ib*	0(0%)	1(2.5%)
	*Ant(2*′*)-Ia*	0(0%)	0(0%)
	*Ant(3*′*)-Ia*	0(0%)	0(0%)
rRNA methyltransferases (RMT)	*ArmA*	0(0%)	3(7.5%)
	*RmtA*	0(0%)	0(0%)
	*RmtB*	44(52.4%)	22(55%)
	*RmtC*	0(0%)	0(0%)
	*RmtD*	0(0%)	0(0%)
	*NpmA*	0(0%)	0(0%)

Notably, one CR-non-hvKp isolate (KpApr172) was resistant to apramycin. PCR showed that it contains the aminoglycoside acetyltransferase 3-IV gene *aac(3)-IV*, which encodes resistance to apramycin, gentamicin, netilmicin and tobramycin. Further next generation sequencing revealed that KpApr172 was a strain ST11 and harbored the KL47 capsular type. Resistance gene mining indicated that KpApr172 harbored 17 antimicrobial resistance genes encoding resistance to β-lactams (*bla*_KPC–2_, *bla*_NDM–5_, *bla*_CTX–M–3_, and *bla*_SHV–12_), aminoglycosides [*armA*, *aadA2*, *aac(3)-IId*, *aac(3)-IV* and *aph(4)-Ia*], macrolid [*msr(E)* and *mph(E)*], bleomycin (*ble*), florfenicol (*floR*), quaternary ammonium compound (*qacEΔ1*) and sulfonamide-trimethoprim (*sul1, sul2* and *dfrA12*). The quinolone resistance-determining region (QRDR) genes *gyrA*, *gyrB*, *parC* and *parE* were further examined, and we observed mutations encoding amino acid substitutions at Ser83-Ile and Asp87-Gly within the QRDR regions of GyrA, and Ser80-Ile in ParC. Examination of the outer membrane protein OmpK35 and OmpK36 genes revealed a premature stop codon at AA63 in OmpK35, due to an adenine (A) deletion mutation at nt 82, and the glycine and aspartic acid insertion at AA134. *In silico* plasmid replicon typing identified five plasmid groups in KpApr172, belonging to incompatibility groups X3, R, FII and ColE-like (*n* = 2), respectively.

**FIGURE 2:**
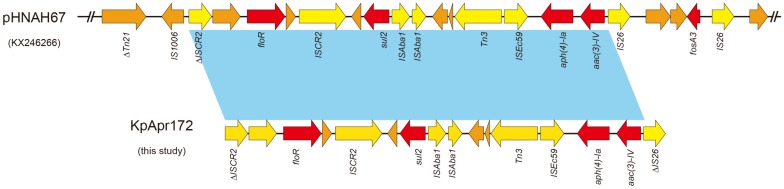
Sequence structure of *aac(3)-IV*-containing element in pHNAH67 (KX246266) and KpApr172 (this study). Colored arrows indicate open reading frames, with red, yellow and orange arrows representing resistance genes, mobile elements and additional genes, respectively. Light blue shading denotes regions of shared 100% homology between different elements.

In order to further characterize the transferability of apramycin resistance gene *aac(3)-IV*, we attempted to transfer them via conjugation to a recipient *E. coli* J53 AzR strain (data not shown), however, we were not able to transfer the *aac(3)-IV* by conjugation, suggesting the gene may be harbored by a non-conjugative plasmid or on the chromosome. Examination of the *aac(3)-IV-*containing contig from the genome assemblies revealed that *aac(3)-IV* was located on a 13,397 bp segment, along with the resistance genes, *floR*, *sul2* and *aph(4)-Ia* (Figure 2). BLASTn analysis showed that the sequences of this contig were identical (100% query coverage and 100% identities) to the corresponding regions in some plasmids, including pHNAH67 (KX246266, from *E. coli* of chicken), pCFSA122-1 (CP033224, from *Salmonella enterica of* pork), pSH16G4466 (MK477617, from *Salmonella enterica* in human), pXGE1mcr (KY990887, from *E. coli* of cow feces), pHNSHP45-2 (KU341381, from *E coli* of pig feces), and chromosome of *E. coli* YSP8-1 (CP037910, from pig feces) from animal or human sources in China.

## Discussion

Aminoglycosides resistance is mostly due to the chemical modification by aminoglycoside-modifying enzymes (AMEs), including acetyltransferases (AACs), phosphotransferases (APHs) and nucleotidyltransferases (ANTs) (Ramirez and Tolmasky, 2010). Some studies had shown AAC(6′)-Ib is one of the most prevalent and clinically relevant AMEs, which confers the resistance to amikacin and other aminoglycosides (Ramirez et al., 2013). A Greek study showed more than 70% of carbapenemases-producing Enterobacteriaceae strains carry *aac(6*′*)-Ib* gene (Galani et al., 2019). However, only 7.14% of CR-hvKp and 7.5% of CR-non-hvKp isolates were found to carry this gene in our study. By contrast, we found a high frequency of ARM/RMT methyltransferases gene in our study. ARM/RMT methyltransferases were found to confer high levels of resistance to most aminoglycosides other than apramycin (Galimand et al., 2003). ARM/RMT genes *armA* and *rmt* had been frequently described in clinical isolates throughout America, Europe and India (Fritsche et al., 2008). Our study showed 93.6% (44/47) CR-hvKp and 95.6% (22/23) CR-non-hvKp amikacin or gentamycin resistant strains contained the 16S rRNA methylase gene *rmtB*. These results suggested that gentamycin and amikacin have limited activities against clinical CRKp isolates, including CR-hvKp, in Chinese hospitals.

In comparison, our results showed that all CR-hvKp isolates were susceptible to apramycin, whereas ∼ 50% of them were resistant to amikacin or gentamycin. Similarly, almost all CR-non-hvKp strains (39/40) were susceptible to apramycin irrespective of the presence of KPC-2 or NDM carbapenemase genes. In addition, our results were in accordance with previous studies that apramycin is not be inactivated by general AMEs and methylases (Livermore et al., 2011), making it an potential antibiotic against clinical amikacin or gentamycin resistant strains.

Nevertheless, apramycin resistance has also been described in clinical isolates worldwide (Threlfall et al., 1986; Chaslus-Dancla et al., 1989, 1991; Hunter et al., 1993; Johnson et al., 1995; Mathew et al., 2003; Zhang et al., 2009). Production of the acetylase *aac(3*′*)-IV* was the major mechanism underlying apramycin resistance in clinical isolates. The *aac(3*′*)-IV* gene encoding apramycin resistance was usually harbored by a mobile plasmid (Chaslus-Dancla et al., 1989; Zhang et al., 2009). In this study, we identified one *aac(3*′*)-IV-*harboring apramycin resistant isolate KpApr172 in CR-non-hvKp with a high MIC value of >128 μg/ml. Genomic analysis showed that KpApr172 belonged to the epidemic CRKp ST11 clone, while the *aac(3*′*)-IV*-harboring contigs showed highly identity to plasmid or chromosome sequences of isolates from animal sources. Since apramycin is only licensed for use in farm animals in China, we suspected that horizontal transfer of apramycin resistant genes from animals to human might be responsible for the dissemination of apramycin resistance (Yates et al., 2004). Therefore, *aac(3*′*)-IV* gene should be preferably screened for apramycin resistant isolates in clinical settings.

In this study no CR-hvKp isolates were found to be resistant to the “last resort” antimicrobial agents, including ceftazidime-avibactam, colistin and tigecycline, however, the treatment arsenal for CR-hvKp could be easily compromised by the spread of mobile resistant genes. Plasmid-mediated colistin and tigecycline resistance mechanisms, MCR-1 and Tet(x), have been described in China (Liu et al., 2016; He et al., 2019; Sun et al., 2019). In addition, the FDA approved cefetazidime-avibactam is ineffective against metallo-β-lactamase (e.g., NDM) producers. Furthermore, the original KPC-2 ST11 CR-hvKp outbreak study showed that despite being *in vitro* susceptible to tigecycline and colistin, long-term treatment with colistin or tigecycline (alone or in combination with several other antibiotics) was not able to eradicate KPC-2-producing ST11 CR-hvKp isolates, resulting in fatal outcomes (Gu et al., 2018). This convergence of increased resistance and hypervirulence of CR-hvKp underscores the need for the improvement of the antimicrobial treatment choices.

Our study showed apramycin had a potent *in vitro* activity to CR-hvKp irrespective of most commonly occurring carbapenemases and aminoglycoside modifying enzymes. In addition, recent studies had showed that apramycin was associated with fewer ototoxic and nephrotoxic side effects in comparison with other human use aminoglycosides antibiotics (Perzynski et al., 1979; Matt et al., 2012; Ishikawa et al., 2019). These findings suggested apramycin and apramycin-derivative compounds could be a promising antibiotic for the treatment of CR-hvKp infections.

To our knowledge, this is the first study to examine the activities of apramycin against CR-hvKp clinical isolates. Our results indicate that apramycin remain highly active against CR-hvKp. These data support a possible role for apramycin in the treatment of infections due to this hypervirulent and multidrug-resistant strains. A further clinical evaluation of apramycin treatment efficacy is warranted.

## Data Availability Statement

The whole-genome sequence of KpApr172 was deposited in the GenBank database under accession number WUJI00000000.

## Author Contributions

MH, XS, and JL contributed to work, data analysis, and manuscript preparation. SN, SC, HD, and FY analyzed the data. Y-WT and BK prepared the manuscript. HZ and LC contributed to study design, data analysis, and manuscript preparation.

## Conflict of Interest

The authors declare that the research was conducted in the absence of any commercial or financial relationships that could be construed as a potential conflict of interest.

## Supplementary Material

FIGURE S1 Dendrogram of PFGE patterns of 15 CR-hvKp isolates from hospitals in Shanghai (a), Suzhou (b), and Jinan (c), respectively. PFGE typing revealed most of the CR-hvKp strains not belong to the same clone.

TABLE S1 Primers used in this study.
