# Supplementary figures and images for: In vitro Activity of Apramycin Against Carbapenem-Resistant and Hypervirulent Klebsiella pneumoniae Isolates
Source: Front Microbiol. 2020 Mar 13;11:425. doi: 10.3389/fmicb.2020.00425 (PMC7083131; doi:10.3389/fmicb.2020.00425)

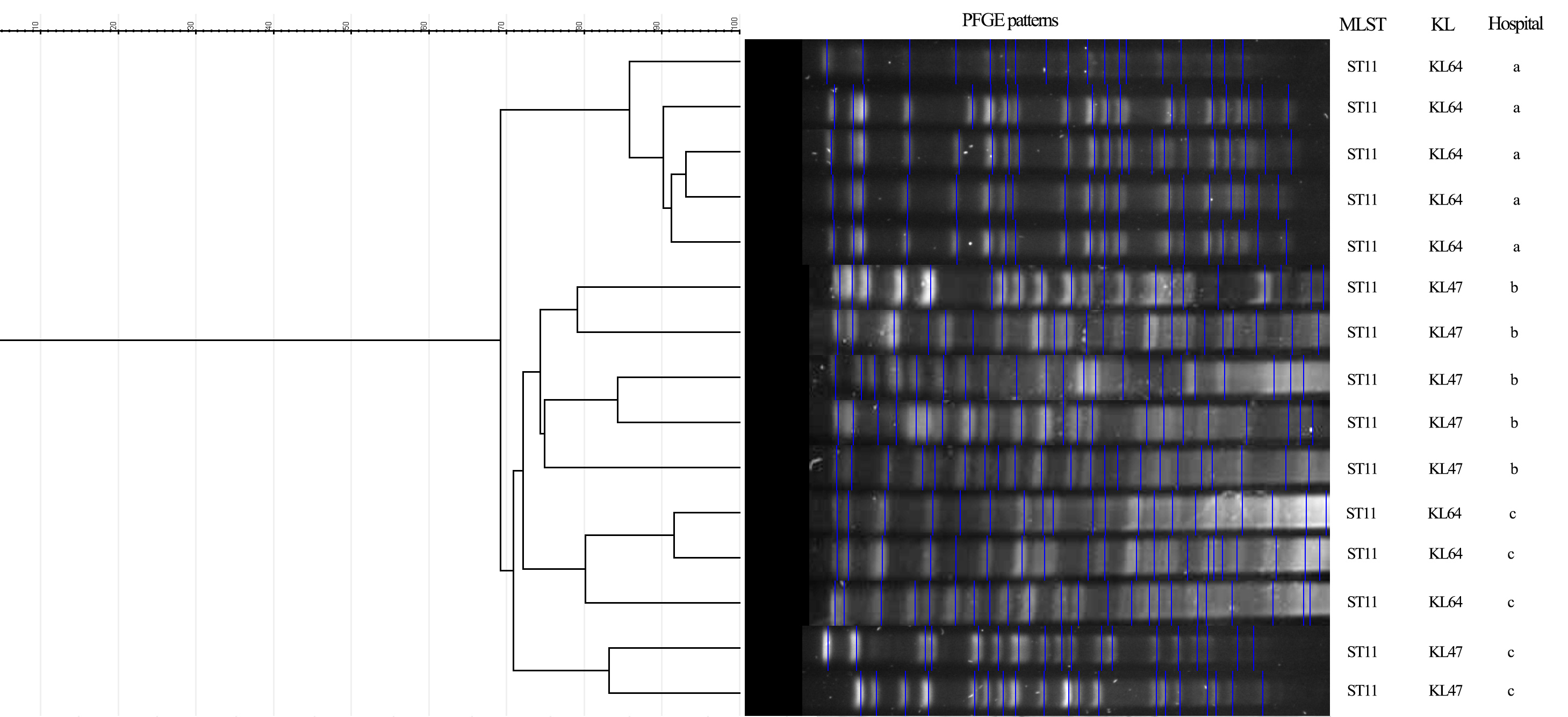

Supplement: FIGURE S1 — Dendrogram of PFGE patterns of 15 CR-hvKp isolates from hospitals in Shanghai (a), Suzhou (b), and Jinan (c), respectively. PFGE typing revealed most of the CR-hvKp strains not belong to the same clone. [file Image_1.jpg]
