# Supplementary material for: In vitro Activity of Apramycin Against Carbapenem-Resistant and Hypervirulent Klebsiella pneumoniae Isolates
Source: Front Microbiol. 2020 Mar 13;11:425. doi: 10.3389/fmicb.2020.00425 (PMC7083131; doi:10.3389/fmicb.2020.00425)
Supplement: TABLE S1 — Primers used in this study. [file Table_1.doc]

Supplementary Table 1. Primers used in this study.

| **Target genes** | **Primer sequence (5’→3’)** | | **Product length (bp)** |
| --- | --- | --- | --- |
| Multilocus sequence typing | | | |
| *rpoB* | F: | GGCGAAATGGCWGAGAACCA | 501 |
|  | R: | GAGTCTTCGAAGTTGTAACC |  |
| *gapA* | F: | TGAAATATGACTCCACTCACGG | 450 |
|  | R: | CTTCAGAAGCGGCTTTGATGGCTT |  |
| *mdh* | F: | CCCAACTCGCTTCAGGTTCAG | 477 |
|  | R: | CCGTTTTTCCCCAGCAGCAG |  |
| *pgi* | F: | GAGAAAAACCTGCCTGTACTGCTGGC | 432 |
|  | R: | CGCGCCACGCTTTATAGCGGTTAAT |  |
| *phoE* | F: | ACCTACCGCAACACCGACTTCTTCGG | 420 |
|  | R: | TGATCAGAACTGGTAGGTGAT |  |
| *infB* | F: | CTCGCTGCTGGACTATATTCG | 318 |
|  | R: | CGCTTTCAGCTCAAGAACTTC |  |
| *tonB* | F: | CTTTATACCTCGGTACATCAGGTT | 414 |
|  | R: | ATTCGCCGGCTGRGCRGAGAG |  |
| Carbapenemase genes | | | |
| *bla*_KPC_ | F: | ATGTCACTGTATCGCCGTCT | 892 |
|  | R: | TTTTCAGAGCCTTACTGCCC |  |
| *bla*_NDM-1_ | F: | CAGCACACTTCCTATCTC | 292 |
|  | R: | CCGCAACCATCCCCTCTT |  |
| *bla*_IMP_ | F: | CATGGTTTGGTGGTTCTTGT | 488 |
|  | R: | ATAATTTGGCGGACTTTGGC |  |
| *bla*_VIM_ | F: | AGTGGTGAGTATCCGACAG | 212 |
|  | R: | TCAATCTCCGCGAGAAG |  |
| *bla*_OXA-48_ | F: | TTGGTGGCATCGATTATCGG | 744 |
|  | R: | GAGCACTTCTTTTGTGATGGC |  |
| AME and RMT-encoding genes | | | |
| *Aac(3’)-II* | F: | ATATCGCGATGCATACGCGG | 877 |
|  | R: | GACGGCCTCTAACCGGAAGG |  |
| *Aac(3’)-IV* | F: | GTCGTCCAATACGAATGGCG | 836 |
|  | R: | CAGCAATCAGCGCGACCTTG |  |
| *Aac(6’)-Ib* | F: | TTGCGATGCTCTATGAGTGGCTA | 472 |
|  | R: | CTCGAATGCCTGGCGTGTTT |  |
| *Aac(6’)-IIa* | F: | CGACCATTTCATGTCC | 542 |
|  | R: | GAAGGCTTGTCGTGTTT |  |
| *Aph(3’)-I* | F: | ATGTGCCATATTCAACGGGAAACG | 816 |
|  | R: | TCAGAAAAACTCATCGAGCATCAA |  |
| *Ant(2’)-Ia* | F: | GCTCACGCAACTGGTCCAGA | 719 |
|  | R: | GGCACGCAAGACCTCAACCT |  |
| *Ant(3’)-Ia* | F: | CATCATGAGGGAAGCGGTG | 787 |
|  | R: | GACTACCTTGGTGATCTCG |  |
| *ArmA* | F: | ATGGATAAGAATGATGTTGTTAAG | 774 |
|  | R: | TTATTTCTGAAATCCACTAGTAATTA |  |
| *RmtA* | F: | ACTGTGATGGGATACGCGTC | 315 |
|  | R: | AGCGATATCCAACACACGATGG |  |
| *RmtB* | F: | ATGAACATCAACGATGCCCTC | 756 |
|  | R: | TTATCCATTCTTTTTTATCAAGTATAT |  |
| *RmtC* | F: | ATGAAAACCAACGATAATTATC | 846 |
|  | R: | TTACAATCTCGATACGATAAAATAC |  |
| *RmtD* | F: | ATGAGCGAACTGAAGGAAAAACTGCT | 744 |
|  | R: | TCATTTTCGTTTCAGCACGTAAAACAG |  |
| *NpmA* | F: | TTGGGTACTGGAGACGGTAG | 421 |
|  | R: | CAGCTTTGTATTGTTCGCTC |  |
| Wzi Gene Sequencing | | | |
| *wzi* | F: | GTGCCGCGAGCGCTTTCTATCTTGGTATTCC | 580 |
|  | R: | GAGAGCCACTGGTTCCAGAACTTCACCGC |  |
| Hypervirulence related genes | | | |
| *rmpA* | F: | ATGTGGCTTGACGTTTCGGGGG | 160 |
|  | R: | GCCGTGGATAATGGTTTACAATTCGGC |  |
| *rmpA2* | F: | GGATGTGGCTTGACATTTCGGGGG | 227 |
|  | R: | TTCATGGATGCCCTCCCTCCTG |  |
| *iutA* | F: | AATCACCTGGGGGCTGGATGCT | 683 |
|  | R: | CCGCACCTTCCACGCCGTAAAT |  |
